# Polygenic scores and Mendelian randomization identify plasma proteins causally implicated in Alzheimer’s disease

**DOI:** 10.3389/fnins.2024.1404377

**Published:** 2024-07-23

**Authors:** Davis B. Cammann, Yimei Lu, Jerome I. Rotter, Alexis C. Wood, Jingchun Chen

**Affiliations:** ^1^Nevada Institute of Personalized Medicine, University of Nevada Las Vegas, Las Vegas, NV, United States; ^2^The Lundquist Institute for Biomedical Innovation, Harbor-UCLA Medical Center, Torrance, CA, United States; ^3^Baylor College of Medicine, Houston, TX, United States

**Keywords:** polygenic score, Mendelian randomization, Alzheimer’s disease, plasma proteins, pQTL

## Abstract

**Background:**

An increasing body of evidence suggests that neuroinflammation is one of the key drivers of late-onset Alzheimer’s disease (LOAD) pathology. Due to the increased permeability of the blood–brain barrier (BBB) in older adults, peripheral plasma proteins can infiltrate the central nervous system (CNS) and drive neuroinflammation through interactions with neurons and glial cells. Because these inflammatory factors are heritable, a greater understanding of their genetic relationship with LOAD could identify new biomarkers that contribute to LOAD pathology or offer protection against it.

**Methods:**

We used a genome-wide association study (GWAS) of 90 different plasma proteins (*n* = 17,747) to create polygenic scores (PGSs) in an independent discovery (cases = 1,852 and controls = 1,990) and replication (cases = 799 and controls = 778) cohort. Multivariate logistic regression was used to associate the plasma protein PGSs with LOAD diagnosis while controlling for age, sex, principal components 1–2, and the number of *APOE*-e4 alleles as covariates. After meta-analyzing the PGS-LOAD associations between the two cohorts, we then performed a two-sample Mendelian randomization (MR) analysis using the summary statistics of significant plasma protein level PGSs in the meta-analysis as an exposure, and a GWAS of clinically diagnosed LOAD (cases = 21,982, controls = 41,944) as an outcome to explore possible causal relationships between the two.

**Results:**

We identified four plasma protein level PGSs that were significantly associated (FDR-adjusted *p* < 0.05) with LOAD in a meta-analysis of the discovery and replication cohorts: CX3CL1, hepatocyte growth factor (HGF), TIE2, and matrix metalloproteinase-3 (MMP-3). When these four plasma proteins were used as exposures in MR with LOAD liability as the outcome, plasma levels of HGF were inferred to have a negative causal relationship with the disease when single-nucleotide polymorphisms (SNPs) used as instrumental variables were not restricted to cis-variants (OR/95%CI = 0.945/0.906–0.984, *p* = 0.005).

**Conclusion:**

Our results show that plasma HGF has a negative causal relationship with LOAD liability that is driven by pleiotropic SNPs possibly involved in other pathways. These findings suggest a low transferability between PGS and MR approaches, and future research should explore ways in which LOAD and the plasma proteome may interact.

## 1 Introduction

Late-onset Alzheimer’s disease (LOAD) is a progressive, neurodegenerative condition with no known cure and a diverse range of contributing pathologies that make it difficult to diagnose (Fang et al., 2020). Even after diagnosis, this treatment is hindered because LOAD patients often exhibit heterogeneity in their clinical symptoms at diagnosis (Devi and Scheltens, 2018), brain neuropathology (Murray et al., 2011), and comorbidities from other diseases such as type 2 diabetes (Santiago and Potashkin, 2021). Therefore, they likely require therapeutic options tailored to their individual needs. Due to the highly polygenic nature of LOAD, there are many potential contributing genetic factors to either disease risk or protection against it, none of which are individually necessary or sufficient for the development of LOAD (Baker and Escott-Price, 2020). Furthermore, many of the genes contributing to the risk of LOAD are considered to be pleiotropic, with upstream effects on multiple different traits that may give rise to some of the patterns of comorbidities seen in the LOAD patient population. Understanding the polygenic overlap of different traits with LOAD could help guide diagnosis and treatment by pinpointing which factors negatively or positively contribute to the overall health of patients, thus aiding in the management of risks.

Like LOAD, circulating levels of cytokines and other plasma proteins are highly heritable and polygenic (de Craen et al., 2005; Li et al., 2016). Blood samples from children of parents with a history of LOAD show a higher production capacity for multiple pro-inflammatory cytokines than those from children without a familial history of LOAD, suggesting a shared genetic liability between inflammation and LOAD (van Exel et al., 2009). This notion is supported by recent genome-wide association studies (GWASs) for LOAD, in which several associated loci were implicated in inflammatory pathways (Harold et al., 2009) and candidate gene investigations (Desikan et al., 2015). Genetic risk factors for LOAD, including the *APOE*-e4 isoform and rare variants of *TREM2*, have further been shown to exacerbate neuroinflammation through their effect on the activation state of microglia (Colonna and Wang, 2016; Parhizkar and Holtzman, 2022). Recent studies have also suggested a genetic overlap between predictors of circulating proteins and LOAD, with some exploring inflammation-specific markers (van der Linden et al., 2021a) and others focusing on plasma proteins with evidence of a role in the disease (Handy et al., 2021).

The understanding of polygenic traits and their relationship with other phenotypes has been considerably improved by the implementation of polygenic scores (PGSs), which are single-unit estimates of an individual’s genetic liability for a trait (Dudbridge, 2013). PGSs represent the sum of individual predisposing single-nucleotide polymorphisms (SNPs), which tend to be weighted by their effect size as drawn from a GWAS. A PGS is typically calculated using individual genotyping data in a target population according to the effect allele of SNPs and their effect sizes provided by GWAS summary statistics. PGSs are most commonly developed for disease prediction, with genotyping information and GWAS summary statistics for the same trait. For example, LOAD-specific PGSs have achieved an area under the curve of up to 84% in distinguishing LOAD cases from controls (Leonenko et al., 2021). Using summary statistics of a trait different from the target trait provides a measure of shared genetic etiology between the two (Choi et al., 2020). This approach has seen recent success in identifying genetic associations between the blood levels of 31 different lipids consistent across two independent LOAD target cohorts (van der Linden et al., 2021b) and in showing associations between a LOAD PGS and markers of inflammation (Morgan et al., 2017). However, as shown by Handy et al. (2021), using a GWAS of plasma proteins to create PGSs of LOAD for association testing requires further causal validation, as even literature-selected proteins may show a weak association with the disease after accounting for factors such as population stratification.

A genetic association does not necessarily indicate that a trait causally contributes to disease pathology, as even at the genetic level, associations are subject to confounding. Mendelian randomization (MR) provides a means by which a causal relationship can be inferred between heritable traits by using SNPs as genetic instruments to test for a causal effect (Davey Smith and Ebrahim, 2003; Davey Smith and Hemani, 2014). In MR, a causal relationship between an exposure and an outcome is inferred where SNPs associated with an exposure trait, e.g., plasma protein levels, show a proportional association with an outcome trait, e.g., LOAD, under the assumption that they do not have an independent impact on the outcome (i.e., no pleiotropic effects across exposure and outcome), and are not associated with confounding variables (Davey Smith and Hemani, 2014). Two-sample MR leverages the MR approach using the summary statistics of two independent GWASs to serve as the exposure and outcome traits. For example, using well-powered GWASs across several measures of body mass, one study found a protective relationship between genetic predictors of lean body mass and LOAD (Daghlas et al., 2023), implicating a causal effect.

Using a recent GWAS of 90 different plasma proteins published by the SCALLOP consortium (Folkersen et al., 2020), we sought to develop plasma protein PGSs and test their association with LOAD diagnosis as a means of identifying novel genetic etiologies and potential pathways that may be associated with the disease. We then selected plasma protein PGSs that showed significant associations with LOAD as exposures in a two-sample MR analysis where LOAD liability served as the outcome to test whether they play a causal role in the disease. We expect that our unbiased selection of proteins and the larger participant sample size (*n* = 17,747) of the plasma protein GWAS used compared to prior studies will aid our goal of discovering novel relationships between plasma proteins and LOAD, which may assist future efforts in identifying diagnostic factors for the disease.

## 2 Materials and methods

### 2.1 Study design

**Figure 1:**
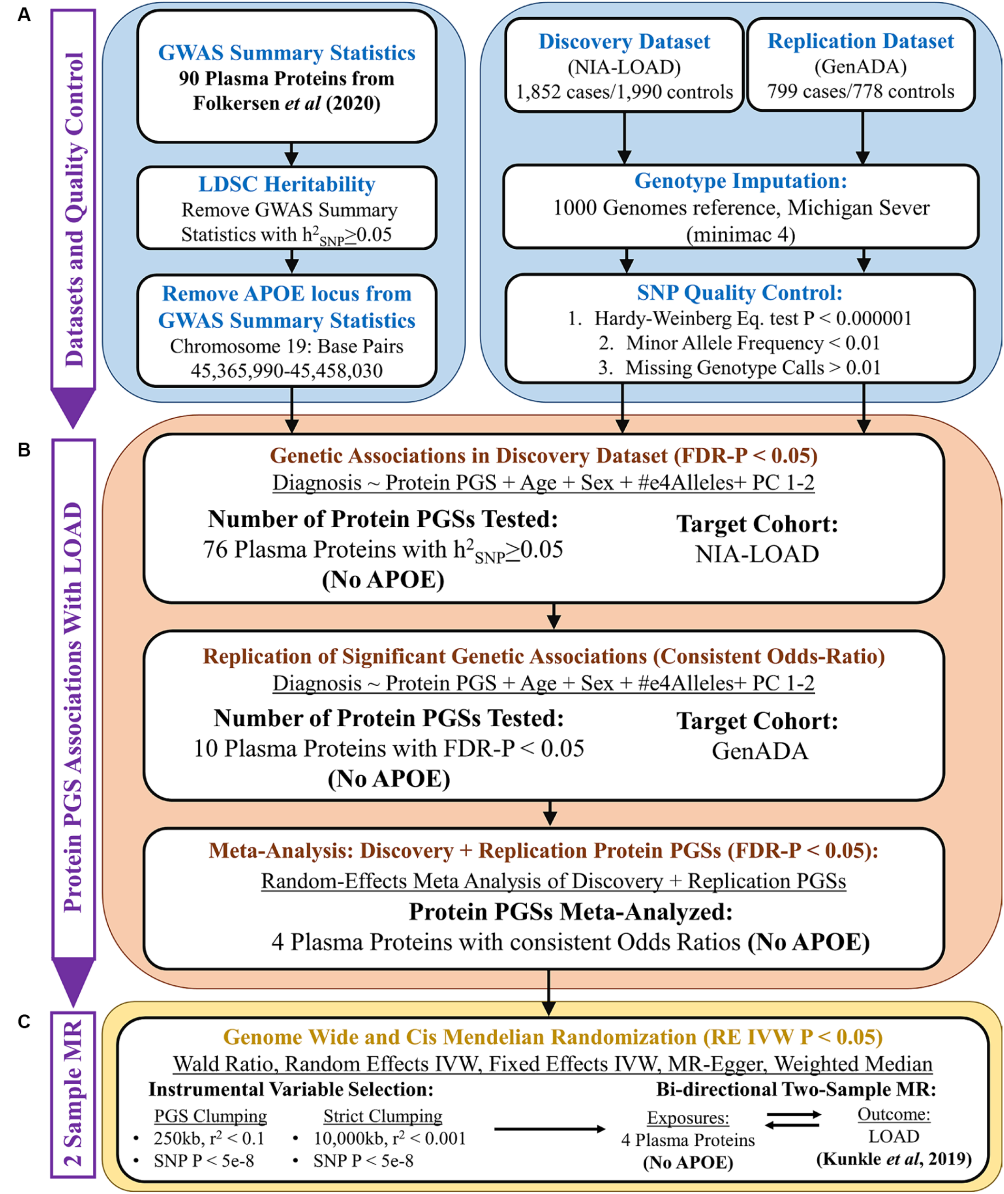
Overall study design. **(A)** Quality control of GWAS summary statistics (base data) and AD genotyping datasets (target data). **(B)** PGS association analysis using PRSice-2, followed by a random-effects meta-analysis. **(C)** Two-sample Mendelian randomization analysis.

The overall design of this study is given in Figure 1. The goal of this study was to discover plasma proteins with a genetic association with LOAD diagnosis using PGSs and then determine if any of them have a causal relationship with LOAD using two-sample MR. To do this, we first performed quality control on the GWAS summary statistics of plasma protein levels, using LDSC to calculate the heritability of each plasma protein and retain summary statistics with an *h*^2^_SNP_ ≥ 0.05. To control the known strong effects of the *APOE* locus, we removed it from the GWAS summary statistics prior to their use in creating plasma proteins PGSs and as exposures in MR (Figure 1A).

Next, PGSs were calculated in our discovery cohort using the summary statistics of plasma proteins (Figure 1B). Plasma protein PGSs that had a significant association (FDR-adjusted *p* < 0.05) with LOAD diagnosis while accounting for the age, sex, *APOE*-e4 genotype, and first two genetic principal components (PCs) of discovery cohort participants were tested in our replication cohort. PGS-LOAD associations with a consistent direction of effect by their odds ratio (OR) in the replication cohort were then meta-analyzed with their discovery cohort PGS-LOAD association in a random-effects model.

The GWAS summary statistics of significant plasma protein PGS-LOAD associations in the meta-analysis (FDR-adjusted *p* < 0.05) were used as exposures in a bidirectional two-sample MR analysis with LOAD as the outcome (Figure 1C). Plasma proteins with a *p*-value less than 0.05 by the random-effects inverse-variance weighted (RE IVW) or Wald ratio methods, and a consistent direction of effect in sensitivity analyses (fixed effects IVW, MR-Egger, weighted median), were inferred to have a causal effect on LOAD liability. As an additional sensitivity analysis, we used a LOAD GWAS as the exposure and the prior plasma protein exposures as outcomes to test possible bidirectional relationships between the plasma proteins and LOAD.

### 2.2 Genotyping (target) data

In this study, two LOAD genotyping datasets were requested from the database of Genotypes and Phenotypes (dbGaPs) for PGS association analyses. These include the National Institute of Aging Late-Onset Alzheimer’s Disease (NIA-LOAD) study (phs000168.v2.p2) (Lee et al., 2008) and the Multi-Site Collaborative Study for Genotype-Phenotype Associations in Alzheimer’s Disease (GenADA) (phs000219.v1.p1) (Li et al., 2008; Filippini et al., 2009). Both studies were conducted on European American (EA) individuals, except the NIA-LOAD study, which included a small cohort of African Americans (AA). Because the plasma protein GWASs were conducted in European-origin populations, we restricted our analyses of the NIA-LOAD study to EA individuals identified using principal component analysis (PCA). The NIA-LOAD study was used as our discovery cohort, while the GenADA study was used as our replication cohort. Information on the diagnosis, age, sex, and *APOE*-ε4 allele frequencies of individuals in the NIA-LOAD and GenADA datasets can be found in Table 1.

**Table 1:** Demographic information of genotyping data.

		NIA-LOAD			GenADA		
		Cases	Controls	Total	Cases	Controls	Total
Sample Size		1852	1990	3,842	799	778	1,577
Age (Mean ± SD)		76.7 ± 6.99	70.8 ± 10.8	73.6 ± 9.61	72.2 ± 8.41	73.4 ± 7.92	72.8 ± 8.19
Sex (Male/Female)		645/1207	789/1201	1434/2408	339/460	276/502	615/962
*APOE-*ε4 Alleles, n (%)	0	575 (31.0)	1,303 (65.5)	1878 (48.9)	296 (37.0)	589 (75.7)	885 (56.1)
	1	1,003 (54.2)	634 (31.9)	1,637 (42.6)	397 (49.7)	177 (22.8)	574 (36.4)
	2	274 (14.8)	53 (2.66)	327 (8.51)	106 (13.3)	12 (1.54)	118 (7.48)

Age, age at onset (cases)/age at examination (controls); *APOE*-ε4 alleles, number of ε4 alleles in the genome of each individual.

LOAD cases in both studies were defined as any individual with probable LOAD dementia by the National Institute of Neurological and Communicative Disorders and Stroke and the Alzheimer’s Disease and Related Disorders Association (NINCDS-ADRDA) criteria. Controls were neurologically evaluated to be cognitively normal and matched to the age and sex of cases. NIA-LOAD participants (cases = 1,852 and controls = 1,990) had 601,273 SNPs genotyped using the Illumina Human610 QuadV1-B platform. In the GenADA study, participants (cases = 799 and controls = 778) were genotyped using the Affymetrix 500 k Set, which includes the Mapping 250 k STY and Mapping 250k_NSP arrays. To fill in missing genetic information, both genotyping datasets were imputed to genome build 37 (hg19) with the 1,000 Genomes Phase 3v5 reference panel (Auton et al., 2015) on the Michigan Imputation Server^1^ (Das et al., 2016). After imputation, we used Plink (v.1.9) to quality control SNPs with imputation quality (INFO) greater than 0.3, a minor allele frequency less than 0.01, Hardy–Weinberg equilibrium test *p*-value less than 0.000001, missing genotype rate, and missing rate per person less than 0.01 (Chang et al., 2015), resulting in a final total of 8,530,670 SNPs in both datasets.

### 2.3 GWAS summary statistics

In this study, we used summary statistics from GWASs of plasma levels of proteins and of LOAD. Prior to generating PGSs, we removed all SNPs within the *APOE* locus (genome build hg19-chromosome 19; base pairs 45,365,990–45,458,030) of the plasma protein summary statistics. This is because the *APOE* locus has a large effect on LOAD risk, and including many SNPs in linkage disequilibrium (LD) with *APOE* would falsely tag its effects and potentially bias any PGS results (Farrell and Brookes, 2022). Information on the two studies is summarized in Table 2.

**Table 2:** GWAS summary statistics information.

Author (Year)	Consortia	Trait(s)	Ancestry	Sample Size	PMID
Kunkle et al. (2019)	IGAP	Clinically diagnosed LOAD	European	21,982 Cases, 41,944 Controls	30,820,047
Folkersen et al. (2020)	SCALLOP	90 Plasma Proteins	European	17,747	33,067,605

Author (year) = Primary author of GWAS study and year of study publication, consortia = consortia involved in the conduct of the GWAS study, trait(s) = traits studied by GWAS represented in summary statistics, ancestry = study population ethnicity, sample size = number of study participants, PMID = PubMed ID.

#### 2.3.1 LOAD summary statistics

We downloaded the Stage 12,019 Kunkle et al. (2019) GWAS summary statistics from the IEU GWAS catalog.^2^ The International Genomics of Alzheimer’s Project (IGAP) is a large three-stage study based on GWAS on individuals of European ancestry. In stage 1, IGAP used genotyped and imputed data on 11,480,632 SNPs to meta-analyze GWAS datasets consisting of 21,982 LOAD cases and 41,944 cognitively normal controls from four consortia: The Alzheimer Disease Genetics Consortium (ADGC); The European Alzheimer’s disease Initiative (EADI); The Cohorts for Heart and Aging Research in Genomic Epidemiology Consortium (CHARGE); and The Genetic and Environmental Risk in AD Consortium Genetic and Environmental Risk in AD/Defining Genetic, Polygenic and Environmental Risk for Alzheimer’s Disease Consortium (GERAD/PERADES).

#### 2.3.2 Plasma protein summary statistics

The summary statistics for each of the 90 blood plasma proteins by Folkersen et al. (2020) were downloaded from Zenodo.^3^ To discover genome-wide significant loci for each of the 90 proteins, a meta-analysis was performed on 21.4 million SNPs derived from 13 studies totaling 21,758 European individuals. Due to inter-cohort differences in genotype imputation, each protein had an average sample size of 17,747 individuals with 20.3 million SNPs. In each cohort, blood plasma levels of proteins were measured using the Olink proximity extension assay cardiovascular 1 panel (Assarsson et al., 2014). The log2 normalized protein expression (NPX) values from the Olink assay for each protein had been ranked and either inverse normal transformed or standardized to unit variance to control for batch effects. In total, 467 genome-wide significant loci were reported in the original study to be associated with 85 of the 90 blood plasma proteins.

### 2.4 Heritability estimated from GWAS summary statistics

The heritability (*h*^2^) of a trait is defined as the proportion of its phenotypic variance attributed to genetic variance (Evans et al., 2018). Estimates of heritability ascribed to SNPs (*h*^2^_SNP_) are important in ensuring the reliability of analyses using GWAS data. Following the recommendation by Choi et al. (2020), we required an *h*^2^_SNP_ ≥ 0.05 for each plasma protein GWAS before performing PGS analyses. We calculated the *h*^2^_SNP_ from each GWAS using LD score regression (LDSC) (Bulik-Sullivan et al., 2015). LDSC calculates an “LD Score” for each SNP in a GWAS, which measures the amount of genetic variance tagged by the SNP. The *χ*^2^ association test statistic for each SNP is then regressed against their LD Score, and the slope of this regression serves as an estimate of the GWAS’s *h*^2^_SNP_. Before a GWAS’s *h*^2^_SNP_ calculation, its SNPs were limited to those on an ancestry-matched reference panel of ~1.2 million SNPs from the HapMap 3 project to avoid estimating the *h*^2^_SNP_ with genetic variants of low imputation quality that were not reported by the original GWAS.

### 2.5 Plasma protein PGS modeling

In this study, we generated PGSs with the PRSice-2 software (Choi and O’Reilly, 2019). PRSice-2 takes the “Clumping and Thresholding” (C + T) approach to create a PGS. First, SNPs are grouped across user-defined kilobase (kb) sized regions of the genome, and SNPs in LD above an *r*^2^ threshold are pruned to remove those that are highly correlated (Wray et al., 2014). *p*-values from the GWAS summary statistics are then used to select a set of clumped SNPs under different *p*-value thresholds (*P*_T_), which are then used to generate the PGSs. A PGS is generated as the sum of effect alleles in a target individual’s genome that are weighted by the effect size of those alleles drawn from GWAS summary statistics. We used standardized PGSs of plasma protein levels in our association analysis:


$${PGS}_{j}=\frac{\sum_{i}\left(S_{i}\times G_{ij}\right)-\mathrm{Mean}\left(PGS\right)}{SD\left(PGS\right)}$$

Where *S_i_* is the effect size of the effect allele for SNP *i*, *G_ij_* is the genotype of SNP *i* (0, 1, 2) for individual *j*.

For the C + T approach, we clumped SNPs in 250 kb regions of the genome that had an *r*^2^ greater than 0.1. PGS models for each protein were calculated using the “best-fit” approach implemented in the PRSice-2 program, where a range of *P*_T_s was applied to the base data (the plasma protein GWASs), attempting to find a set of SNPs under a certain *P*_T_ that can explain the most of the target cohort’s phenotype (LOAD diagnosis). In this study, a range of *P*_T_s was assessed from 5 × 10^−8^ to 1 with an incremental interval of 5 × 10^−5^ to find the best PGS model for each protein.

### 2.6 Multivariate logistic regression and meta-analysis

Using PRSice-2, we evaluated the association of our plasma protein PGSs with LOAD diagnosis in a multivariate logistic regression model that included age, sex, *APOE*-ε4 allele genotype, and the first two genetic PCs of the target data individuals as covariates. We used these same covariates when performing the PGS-LOAD association in both the discovery and replication cohort. PGS-LOAD associations were considered significant if their FDR-adjusted *p*-value was less than 0.05. The random-effects meta-analysis between the PGS-LOAD associations of the discovery and replication cohorts was performed using the metafor package in R (Viechtbauer, 2010). PGS-LOAD associations were considered significant in the meta-analysis if their FDR-adjusted summary estimate p-value was less than 0.05.

### 2.7 Mendelian randomization

For our MR analysis, we used the TwoSampleMR (v.0.6.2) package in R (Hemani et al., 2018). MR is used to infer a causal relationship between an exposure trait and outcome trait when a set of SNPs associated with the exposure [referred to as instrumental variables (IVs)] are also associated with the outcome through their effects on the exposure, assuming three key assumptions are met. SNPs used as IVs must be highly associated with the exposure, not associated with traits that confound the exposure or outcome, and not independently associated with the outcome except through the exposure (Davey Smith and Hemani, 2014).

#### 2.7.1 IV selection

For our primary MR analysis, we used the GWAS summary statistics of significant plasma proteins from the PGS meta-analysis as exposures, and a GWAS of clinically diagnosed LOAD as our outcome (Kunkle et al., 2019). In each plasma protein exposure, SNPs used as IVs were genome-wide significant (GWAS *p* < 5 × 10^−8^) and had a first-stage F-statistic greater than 10 to ensure that they were highly associated with the exposure and considered strong IVs (Pierce et al., 2011). To ensure that SNPs were not correlated via LD, we opted to clump them under “strict” parameters (*r*^2^ < 0.001, kb = 10,000) and “PGS” parameters (*r*^2^ < 0.1, kb = 250) that match the default variant clumping strategies of the TwoSampleMR and PRSice-2 software, respectively. This was performed to allow comparison between the methods. For plasma protein exposures, we also tested SNPs under these two clumping strategies in a “Cis” analysis, where only SNPs on the same chromosome as the protein’s original gene were used, and a “Genome-Wide” analysis, where SNPs could come from any chromosome.

#### 2.7.2 Causal effect estimation and sensitivity analyses

To calculate the causal effect of our exposure traits on our outcome, we used the RE IVW or Wald ratio methods as our primary analysis. We used the RE IVW method to match the random-effects meta-analysis done in our PGS association analysis and used the Wald ratio to account for exposures with only one SNP as a valid IV (Bowden et al., 2017; Burgess et al., 2017). We used the fixed effects IVW (FE IVW), MR Egger, and weighted median methods as sensitivity analyses to assess the effects of horizontal pleiotropy and invalid SNPs (Burgess et al., 2013; Bowden et al., 2015, 2016). As an additional analysis to assess potential bidirectional relationships between plasma proteins and LOAD liability, we repeated our two-sample MR analysis using the LOAD GWAS as the exposure and each previously used plasma protein GWAS as the outcome. We considered an exposure to have a significant causal effect on an outcome when its RE IVW or Wald ratio *p*-value was less than 0.05 and its sensitivity analyses had a concordant direction of effect with the primary method.

### 2.8 Ethics approval statement

This study was approved by the University of Nevada Las Vegas (UNLV) Office of Research Integrity (IRB). Informed consent was obtained from all subjects and/or their legal guardian(s) in the contributing studies. Contributing studies received ethical approval from their respective institutional review boards (IRBs).

## 3 Results

### 3.1 Plasma protein PGS associations with LOAD diagnosis

Of the 90 plasma proteins in the original Folkersen et al. GWAS, 76 were identified as sufficiently heritable (*h*^2^_SNP_ ≥ 0.05) for use in the PGS association analysis (Supplementary Table S1). Notably, the plasma protein with the highest *h*^2^_SNP_ in Folkersen et al. was galectin-3 (Gal-3), with its SNPs accounting for an estimated 43.6% of the trait’s heritability by LDSC. After calculating PGSs for each of the 76 viable plasma proteins in our discovery cohort, we found that 10 were significantly associated with LOAD diagnosis (FDR-adjusted *p* < 0.05) (Table 3). Seven of these plasma protein PGSs had a positive association with LOAD diagnosis, including prolactin (PRL), hepatocyte growth factor (HGF), interleukin-1 receptor agonist (IL-1ra), vascular endothelial growth factor D (VEGF-D), interleukin-18 (IL-18), angiopoietin-1 receptor (TIE2), and kidney injury molecule 1 (KIM-1). Three plasma protein PGSs had a negative association with LOAD diagnosis: platelet-derived growth factor subunit B (PDGF-B), matrix metalloproteinase-3 (MMP-3), and fractalkine (CX3CL1). The most significant plasma protein PGS-LOAD association in the discovery cohort was PRL (OR: 1.17, 95%CI: 1.09–1.24, *p*-value: 0.00004).

**Table 3:** Ten plasma protein PGSs from Folkersen et al. were significantly associated with LOAD diagnosis (FDR *p* < 0.05) in the discovery dataset.

Protein	PVT	*R* ^2^	#SNPs	BETA	SE	OR (95%CI)	*p*	FDR
PRL	0.0023	0.0066	2,934	0.155	0.038	1.17 (1.09–1.24)	0.00004	0.003
HGF	0.01395	0.0053	17,367	0.141	0.038	1.15 (1.08–1.23)	0.00021	0.006
IL-1ra	0.00035	0.0052	701	0.138	0.038	1.15 (1.07–1.22)	0.00024	0.006
VEGF-D	0.01	0.0048	13,241	0.133	0.038	1.14 (1.07–1.22)	0.00045	0.009
PDGF-B	0.0036	0.0042	5,655	−0.126	0.039	0.882 (0.806–0.957)	0.00109	0.017
IL-18	0.06145	0.0038	56,531	0.121	0.039	1.13 (1.05–1.2)	0.00174	0.020
TIE2	0.091	0.0038	76,021	0.121	0.039	1.13 (1.05–1.21)	0.00188	0.020
MMP-3	0.0002	0.0034	567	−0.113	0.038	0.893 (0.819–0.968)	0.00293	0.028
CX3CL1	0.0014	0.0031	2,557	−0.107	0.038	0.898 (0.824–0.973)	0.00486	0.041
KIM-1	0.2858	0.0030	164,807	0.109	0.040	1.12 (1.04–1.19)	0.00576	0.044

Plasma protein PGS models generated by Folkersen et al. using multivariate logistic regression in the discovery (NIA-LOAD) cohort (covariates: age, sex, #*APOE*-ε4 alleles, principal components 1 and 2). PVT, “Best” *p*-value threshold from PRSice-2 used to select SNPs from GWAS summary statistics; *R*^2^, Nagelkerke’s pseudo-*R*^2^; #SNPs, number of SNPs included at the PVT; BETA, PGS model coefficient; SE, PGS model standard error; OR (95%CI), odds ratio with 95% confidence interval; *p*, association *p*-value; FDR, false-discovery rate adjusted association *p*-value.

**Figure 2:**
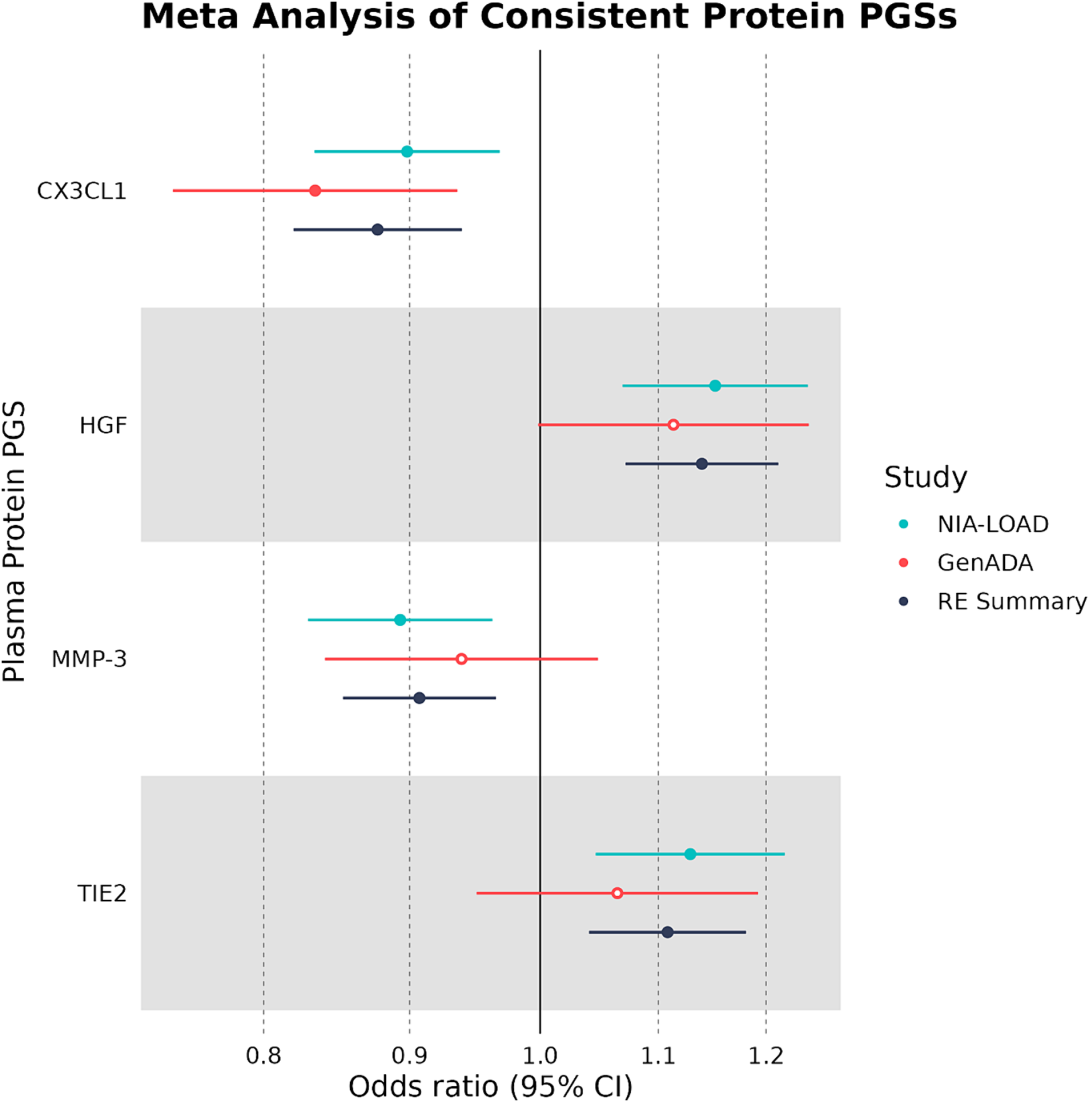
Multivariate logistic regression and meta-analyses of plasma protein PGSs with consistent ORs. Plasma protein PGSs in the discovery (NIA-LOAD) and replication (GenADA) cohorts were meta-analyzed under a random-effects model. Filled-in shapes indicate a significant (FDR-adjusted *p* < 0.05) analysis.

Out of 10 plasma protein PGSs associated with LOAD diagnosis in the discovery cohort, 4 plasma protein PGSs had a consistent OR in the replication cohort (Table 4). Two of these plasma protein PGSs, CX3CL1 and MMP-3, had a consistent negative association with LOAD diagnosis, while HGF and TIE2 had a consistent positive association. Out of all plasma protein PGSs in the replication cohort, only CX3CL1 had a nominally significant association with LOAD diagnosis (OR: 0.842, 95%CI: 0.719–0.949, *p*-value: 0.0019). In a random-effects meta-analysis between the discovery and replication cohort plasma protein PGS-LOAD associations with consistent ORs, all associations remained significant (FDR-adjusted *p* < 0.05) (Figure 2). Overall, this analysis suggested that plasma protein PGSs of CX3CL1 and MMP-3 tended to be higher in control individuals, while PGSs of HGF and TIE2 were higher in individuals diagnosed with LOAD.

**Table 4:** Four plasma protein PGSs with consistent ORs in discovery and replication cohorts.

Protein	Study	PVT	*R* ^2^	BETA	SE	*p*	OR (95% CI)
CX3CL1	**NIA-LOAD**	**0.0014**	**0.0031**	**−0.107**	**0.038**	**0.0049**	**0.898 (0.824–0.973)**
	**GenADA**	**0.0025**	**0.0088**	**−0.182**	**0.059**	**0.0019**	**0.834 (0.719–0.949)**
HGF	NIA-LOAD	0.0140	0.0053	0.141	0.038	0.0002	1.15 (1.08–1.23)
	GenADA	0.0001	0.0034	0.108	0.056	0.0536	1.11 (1–1.22)
MMP-3	NIA-LOAD	0.0002	0.0034	−0.113	0.038	0.0029	0.893 (0.819–0.968)
	GenADA	0.0063	0.0012	−0.063	0.056	0.2593	0.939 (0.828–1.05)
TIE2	NIA-LOAD	0.091	0.0038	0.121	0.039	0.0019	1.13 (1.05–1.21)
	GenADA	0.1225	0.0010	0.062	0.058	0.2821	1.06 (0.951–1.18)

Plasma protein multivariate logistic regression models from Folkersen et al. in discovery (NIA-LOAD) and replication (GenADA) cohorts with consistent odds ratio. PGS models with *p* < 0.05 are bolded. Coefficient, logistic regression model coefficient; SE, standard error; *p*, association *p*-value; OR (95% CI), odds ratio with 95% confidence interval.

### 3.2 Two-sample MR analysis

**Figure 3:**
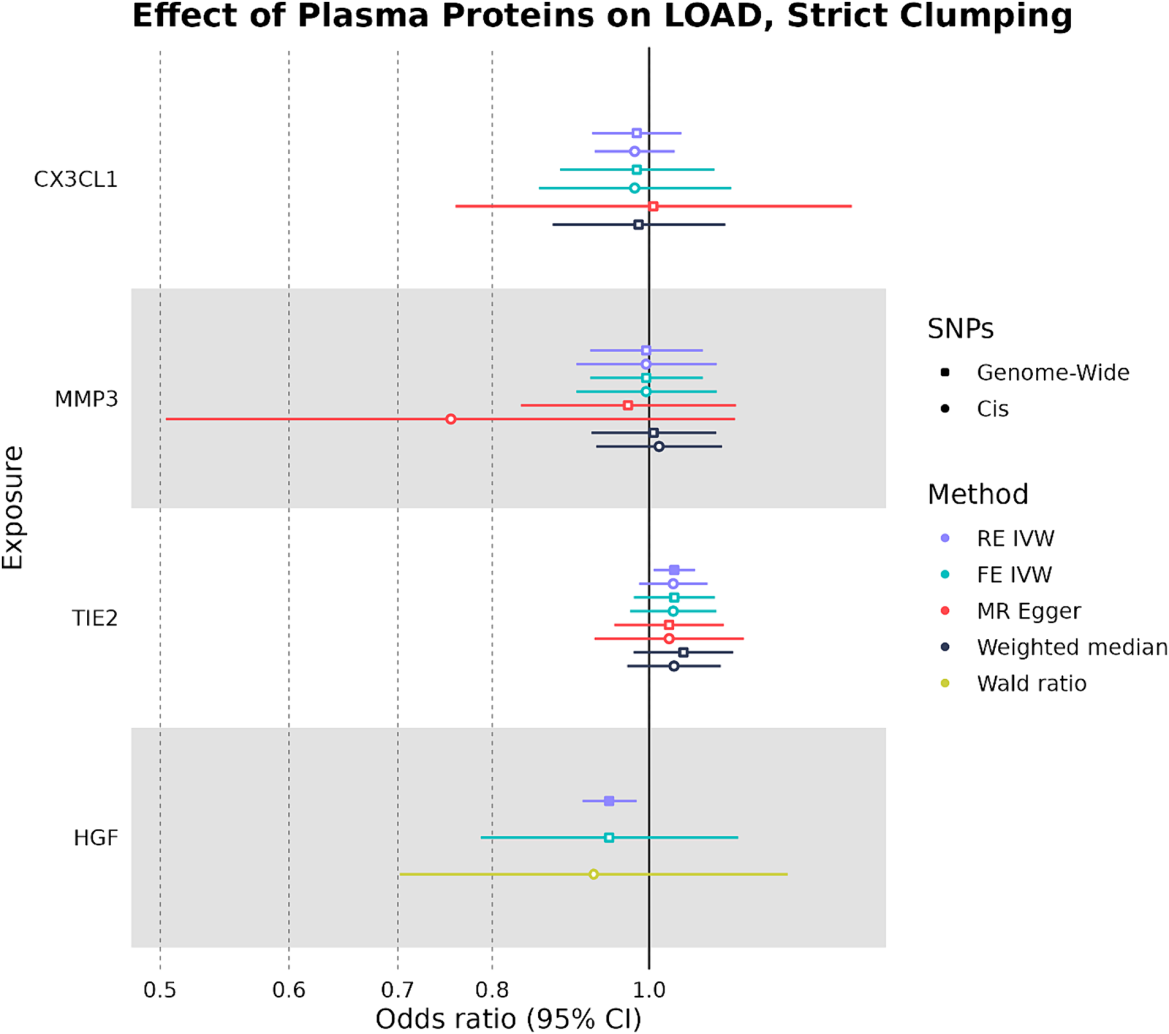
Forest plot of plasma protein-LOAD MR analyses under strict clumping parameters. RE IVW, random-effects inverse-variance weighted analysis and FE IVW, fixed effects inverse-variance weighted analysis. Filled-in shapes indicate a significant (*p* < 0.05) analysis.

We used the four plasma proteins from the random-effects meta-analysis as exposures in two-sample MR to see if any plasma protein had a causal effect on LOAD liability as an outcome. When we selected IVs under strict SNP clumping parameters, two plasma proteins were inferred to have a significant causal effect on LOAD liability, with HGF having a negative causal effect (OR: 0.945, 95%CI: 0.906–0.984, *p*-value: 0.004) and TIE2 having a positive causal effect (OR: 1.04, 95%CI: 1.01–1.07, *p*-value: 0.017) (Figure 3). As plasma HGF had only two valid SNPs for use as IVs in the strict clumping analysis, we were only able to test it under the RE IVW and FE IVW methods, which had a consistent direction of effect (Supplementary Table S3). Notably, plasma TIE2 and HGF were only significant under strict clumping parameters when SNPs used as IVs were sourced genome-wide, as restricting the SNPs to cis-pQTLs on the same chromosome as the plasma protein’s original gene failed to replicate these observed causal effects (Figure 3).

**Figure 4:**
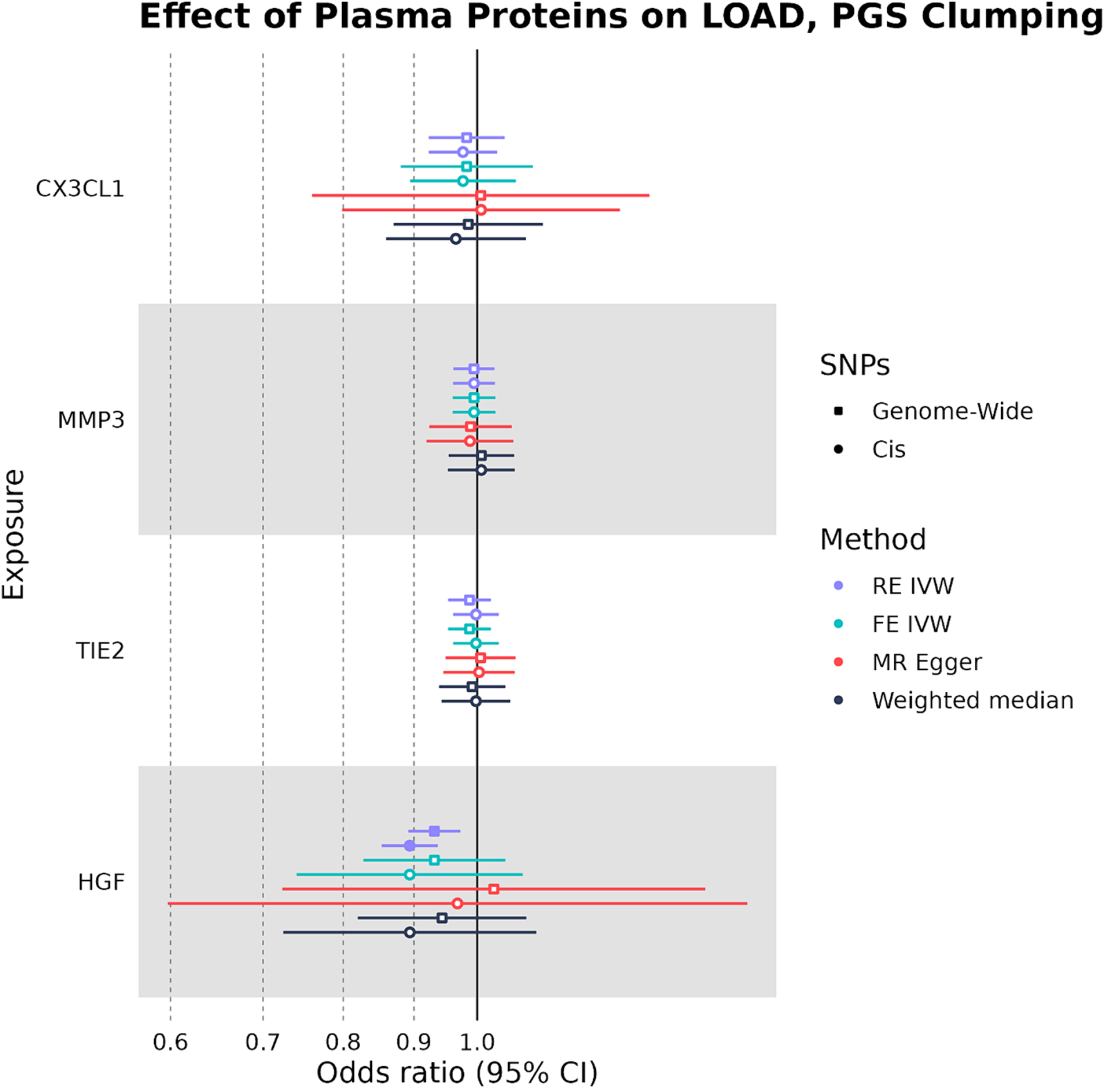
Forest plot of plasma protein-LOAD MR analyses under PGS clumping parameters. RE IVW, random-effects inverse-variance weighted analysis and FE IVW, fixed effects inverse-variance weighted analysis. Filled-in shapes indicate a significant (*p* < 0.05) analysis.

When SNPs were clumped using PGS parameters, plasma HGF had a negative causal effect on LOAD liability that was significant by the RE IVW method in the genome-wide (OR: 0.931, 95%CI: 0.888–0.975, *p*-value: 0.0013) and cis (OR: 0.894, 95%CI: 0.847–0.940, *p*-value: 2.4 × 10^−6^) analyses (Figure 4). While plasma HGF sensitivity analyses had a consistent direction of effect in the cis analysis, the MR-Egger effect estimate of plasma HGF in the genome-wide analysis trended in the opposite direction (OR: 1.03) (Supplementary Table S4). In contrast to the strict clumping parameters, plasma TIE2 was not observed to have a causal effect on LOAD liability when using PGS clumping. Out of the original four plasma proteins used as exposures in both the PGS and strict clumping analyses, plasma HGF was the only one to have a consistent negative causal effect on LOAD liability.

**Figure 5:**
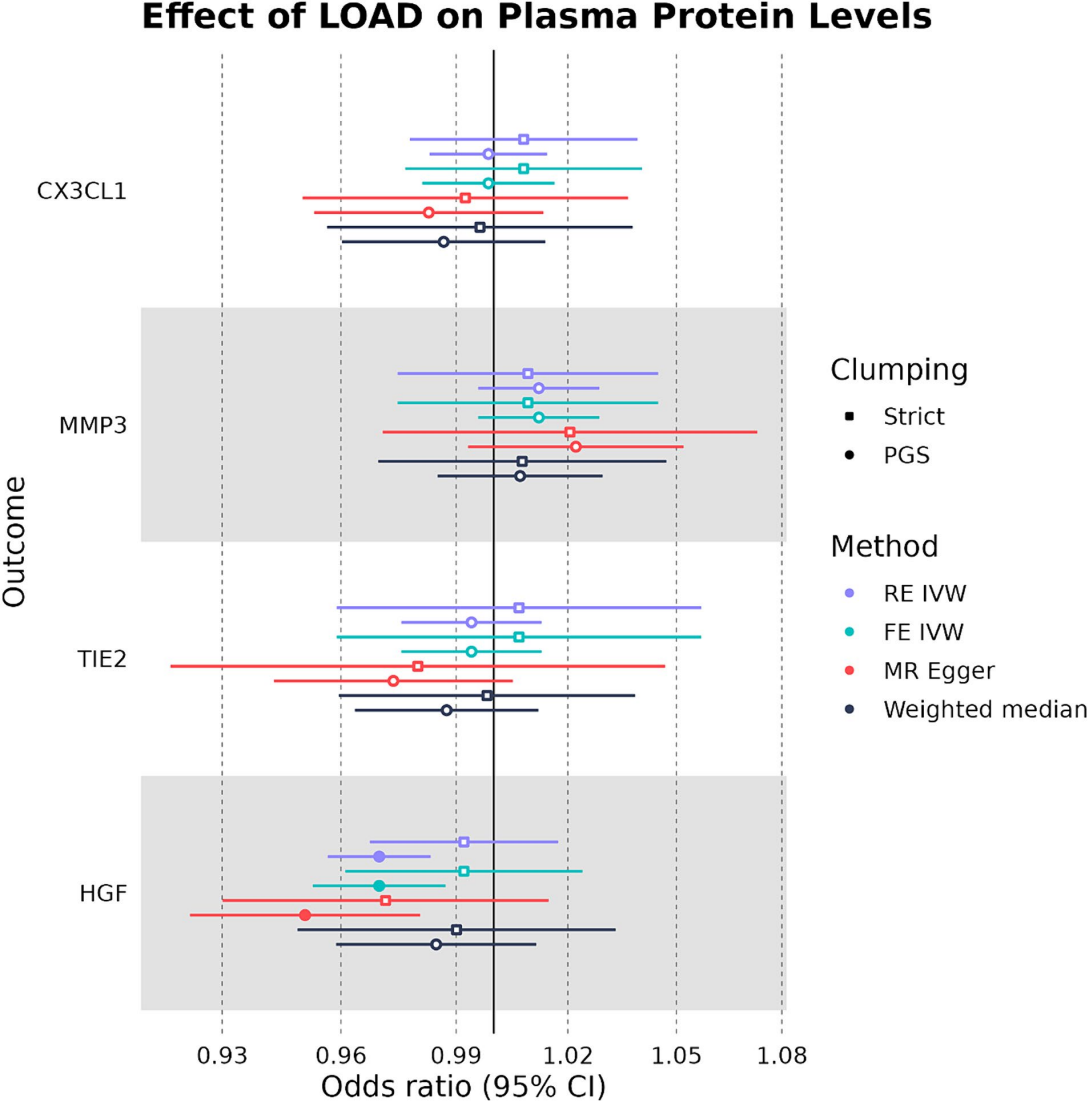
Forest plot of LOAD-plasma protein MR analyses. RE IVW, random-effects inverse-variance weighted analysis and FE IVW, fixed effects inverse-variance weighted analysis. Filled-in shapes indicate a significant (*p* < 0.05) analysis.

In our bidirectional analysis, where LOAD liability was used as an exposure and plasma CX3CL1, HGF, TIE2, and MMP-3 were used as outcomes, we found that LOAD liability had a significant negative causal effect on plasma HGF levels (OR: 0.970, 95%CI: 0.956–0.984, *p*-value: 1.33 × 10^−5^) (Figure 5). In addition to having a consistent direction of effect, the FE IVW (OR: 0.970, 95%CI: 0.952–0.988, *p*-value: 0.00072) and MR-Egger (OR: 0.970, 95%CI: 0.956–0.984, *p*-value: 1.33 × 10^−5^) sensitivity analyses were also significant in the relationship between LOAD liability and plasma HGF (Supplementary Table S5). However, this effect on plasma HGF was only observed when SNPs were clumped with the PGS parameters. Overall, our MR analysis suggests that genetically proxied plasma HGF may exert a slight protective effect against LOAD liability, and in concordance, LOAD liability may have an effect on lowering levels of plasma HGF.

## 4 Discussion

Using GWAS summary statistics of the plasma levels of 76 different proteins (*n* = 17,747), we identified four plasma protein PGSs that had a significant association with LOAD diagnosis across both an independent discovery (cases = 1,852 and controls = 1,990) and replication (cases = 799 and controls = 778) cohort. Of these four plasma protein PGSs, CX3CL1 and MMP-3 had a negative association with the LOAD diagnosis, implicating a protective relationship. Plasma HGF and TIE2 PGSs had a positive association with LOAD diagnosis, suggesting a risk-factor relationship. Using two-sample MR, we found no bidirectional causal relationship between CX3CL1, MMP-3, or TIE2 and LOAD liability. We inferred a protective causal relationship between plasma HGF and LOAD liability when SNPs used as IVs were not restricted to the same chromosome as the HGF gene. Cis-IV selection for plasma HGF exposure only became significant when SNP clumping parameters were relaxed to those used during the PGS association analysis. Using this same clumping strategy, we also identified a negative causal effect of LOAD liability on plasma HGF levels, but not when the strict parameters were used to clump SNPs.

Combining plasma protein GWAS summary statistics from Folkersen et al. with LOAD genotyping from two independent cohorts, we identified four plasma proteins that were consistently associated with LOAD diagnosis through PGSs but did not translate into a consistent causal relationship between the levels of the plasma proteins and one’s genetic liability for LOAD. Notably, plasma HGF PGSs had a positive association with LOAD diagnosis, but plasma HGF levels as an exposure were found to have a negative causal relationship with LOAD liability as an outcome in two-sample MR. HGF itself is a highly pleiotropic cytokine with functions across the body and CNS and is believed to play a role in the regulation of adult brain plasticity and learning (Shimamura et al., 2006; Kato et al., 2012; Desole et al., 2021). However, a prior MR investigation using plasma HGF as an exposure against LOAD and hippocampal volume outcomes found no evidence of a causal effect on either outcome using the IVW method, which is consistent with our findings using this same approach (Fani et al., 2021). HGF levels in the cerebrospinal fluid (CSF) have also shown positive correlations with mild cognitive impairment and other LOAD biomarkers in an observational study, further suggesting that any observed protective function of plasma HGF in LOAD may be pleiotropic rather than a direct effect on the brain (Zhao et al., 2021). Given that plasma HGF as exposure was significant in our study when selecting genome-wide rather than cis SNPs under the RE IVW method, which is adjusted for possible pleiotropy, this could explain the observed causal effect (Bowden et al., 2017).

Our study has several strengths and limitations. A strength of our approach is the use of an independent discovery and replication cohort and meta-analysis to avoid false positives in the PGS association analysis, as well as filtering the GWAS summary statistics by their *h*^2^_SNP_ beforehand to avoid the inclusion of plasma proteins with low SNP-based heritability. Another strength of our study is the use of an orthogonal statistical method, two-sample MR, to confirm results from our PGS analysis. This is because MR employs stricter assumptions for the SNPs proposed to drive an exposure–outcome relationship, most notably the use of SNPs that are significantly associated with the exposure, and the requirement that they do not have pleiotropic effects on the outcome or confounding factors. A potential limitation of developing PGSs for a trait separate from the phenotype of a target cohort is that the PGSs tend to explain a low amount of variance in the target phenotype. Although we were able to identify associations between the PGSs of plasma proteins and LOAD diagnosis that were robust to confounding factors (age, sex, # *APOE*-e4 alleles, and genetic PCs), the PGSs themselves rarely explained more than 1% of the variance in the case/control phenotype. Additionally, we only considered the *APOE*-e4 genotype in our logistic regression models rather than the full *APOE* genotype, which leaves out the known protective effects of the *APOE*-e2 genotype against LOAD liability (Reiman et al., 2020). Other studies using PGSs to associate plasma proteins with LOAD have shown a similar trend in the variance explained by their PGSs (Handy et al., 2021; van der Linden et al., 2021a). This is likely because plasma proteins are involved in a myriad of other pathways unrelated to LOAD, leading to a large possibility of confounding and pleiotropy with a neurodegenerative disease of the CNS (Handy et al., 2021).

The potential for confounding in the plasma protein-LOAD relationship highlights the importance of two-sample MR to control potential false positives that may be influenced by these factors. Due to the aforementioned considerations about using a different “base” and “target” trait when generating PGSs, additional forms of verification are needed to ensure the validity of the association. To improve our approach for future studies, the inclusion of LOAD target cohorts in the PGS analysis with information available on the plasma levels of proteins and metabolites in the participants could help to ensure the validity of the relevant protein PGSs. In addition, the inclusion of more diverse cohorts would improve the generalizability of the results. Future studies that seek to address the genetic relationship between plasma proteins and LOAD should focus on the role of confounders that may affect the interaction of these plasma proteins with the disease due to their role in different pathways. Additionally, future research should seek to understand the underlying mechanisms and pathways by which LOAD may induce changes in the plasma proteome.

## Data availability statement

The summary statistics of the 90 plasma proteins by Folkersen et al are deposited in the Zenodo repository (https://zenodo.org/records/2615265). The summary statistics of the LOAD GWAS by Kunkle et al are deposited in the GWAS catalog, accession number GCST007511. The NIA-LOAD and GenADA genotyping datasets are deposited in the dbGaP database, with accession numbers phs000168.v2.p2 and phs000219.v1.p1 respectively.

## Ethics statement

The studies involving humans were approved by University of Nevada Las Vegas (UNLV) Office of Research Integrity. The studies were conducted in accordance with the local legislation and institutional requirements. Written informed consent for participation was not required from the participants or the participants’ legal guardians/next of kin in accordance with the national legislation and institutional requirements.

## Author contributions

DC: Conceptualization, Data curation, Formal analysis, Investigation, Methodology, Software, Validation, Visualization, Writing – original draft, Writing – review & editing. YL: Data curation, Methodology, Resources, Software, Visualization, Writing – review & editing. JR: Supervision, Writing – review & editing. AW: Supervision, Writing – review & editing. JC: Conceptualization, Data curation, Funding acquisition, Methodology, Project administration, Resources, Supervision, Writing – review & editing.

## Footnotes

^1^ https://imputationserver.sph.umich.edu

^2^ https://www.ebi.ac.uk/gwas/studies/GCST007511

^3^ https://zenodo.org/records/2615265
